# CANTATAdb 3.0: An Updated Repository of Plant Long Non-Coding RNAs

**DOI:** 10.1093/pcp/pcae081

**Published:** 2024-07-17

**Authors:** Michał Wojciech Szcześniak, Elżbieta Wanowska

**Affiliations:** Laboratory of RNA Biology, Institute of Human Biology and Evolution, Adam Mickiewicz University, ul. Uniwersytetu Poznańskiego 6, Poznan 61-614, Poland; Laboratory of RNA Biology, Institute of Human Biology and Evolution, Adam Mickiewicz University, ul. Uniwersytetu Poznańskiego 6, Poznan 61-614, Poland

**Keywords:** Database, lncRNA identification, Long non-coding RNAs, Plant RNAs

## Abstract

CANTATAdb 3.0 is an updated database of plant long non-coding RNAs (lncRNAs), containing 571,688 lncRNAs identified across 108 species, including 100 Magnoliopsida (flowering plants), a significant expansion from the previous version. A notable feature is the inclusion of 112,980 lncRNAs that are expressed specifically in certain plant organs or embryos, indicating their potential role in development and organ-specific processes. In addition, CANTATAdb 3.0 includes 74,886 pairs of evolutionarily conserved lncRNAs found across 47 species and inferred from genome–genome alignments as well as conserved lncRNAs obtained using a similarity search approach in 5,479 species pairs, which would further aid in the selection of lncRNAs for functional studies. Interestingly, we find that conserved lncRNAs with tissue-specific expression patterns tend to occupy the same plant organ across different species, pointing toward conserved biological roles. The database now offers extended search capabilities and downloadable data in popular formats, further facilitating research on plant lncRNAs.

## Introduction

Long non-coding RNAs (lncRNAs) are RNA molecules longer than 200 nucleotides that do not have the ability to encode proteins but are involved in fundamental biological processes and have a wide range of functions. In plants, their complex roles have been observed in different contexts, such as growth and development (Liu et al. 2018) and stress response (Zhao et al. 2023). These functions can be achieved by influencing different stages of gene expression. For example, plant lncRNAs can perform their functions by binding to chromatin. One such example is flowering-associated intergenic lncRNA (FLAIL), which was found to repress flowering in *Arabidopsis*. Jin et al. showed that binding of the FLAIL lncRNA to specific chromatin regions promotes the expression of flowering repressors. In addition, this lncRNA regulates alternative splicing by interacting with the spliceosome complex (Jin et al. 2023). Plant lncRNAs can also act as miRNA decoys or sponges. Yadav et al. screened lncRNAs from three different species for their characteristic features and found endogenous target mimic sites for different miRNAs in all the plants studied, suggesting that this is a common mechanism for fine-tuning miRNA activity in the cellular environment (Yadav et al. 2023). It has also been shown that plant lncRNAs function by binding to other molecules, proteins, DNA or RNA. For example, the alternative splicing competitor lncRNA controls the transcriptional response to flagellin22 and reduces root growth sensitivity to this important signaling molecule by physically interacting with PRP8a and SmD1b, two crucial parts of the spliceosome core, as well as nuclear speckle RNA-binding proteins, which are known to regulate alternative splicing (Rigo et al. 2020).

The increasing number of lncRNAs found in many plant species attests to their role in plant development, growth and stress response, making it essential to deeply understand their properties and functions. With this in mind, we previously created and published CANTATAdb, a database of lncRNAs from 10 model species (Szcześniak et al. 2016). In 2019, we expanded this online resource to 39 species (Szcześniak et al. 2019). Here, we present the updated CANTATAdb database (CANTATAdb 3.0) with 108 species, including six algae. For species already included in the previous releases of CANTATAdb, updated genomes have been applied (if available) and expanded sets of RNA-Seq samples were also used. One of the novelties introduced in CANTATAdb 3.0 is data on evolutionarily conserved lncRNAs, which were inferred from genome–genome alignments (synteny) and independently using a similarity search approach. In addition, CANTATAdb 3.0 includes lncRNAs with plant part or embryo-specific expression. The high expression specificity makes them potentially useful as markers for organs and developmental stages and is likely related to their cellular roles (Zhao et al. 2020). Interestingly, our research has shown that lncRNAs unique to one part of the plant tend to be enriched in the same part in other species, suggesting conserved cellular roles. The data are freely available to search, browse and download at http://cantata.amu.edu.pl and http://yeti.amu.edu.pl/CANTATA/.

## Results and Discussion

### CANTATAdb 3.0 summary statistics

In this updated version, CANTATAdb 3.0 stores 571,688 lncRNAs identified across 108 species, compared to 239,631 lncRNAs for 36 plants and three algae species in its previous version (Szcześniak et al. 2019). In more detail, the database now features 100 Magnoliopsida (flowering plants) species, along with selected representatives of seven additional classes, some of which do not belong to the Viridiplantae kingdom (**Table 1**). Importantly, the particular families have been substantially expanded, with Poaceae now represented by 32 species (previously 12), Brassicaceae by 10 species (previously five) and Fabaceae by 10 species (previously three) (**Supplementary Table S1**). In addition to the types of data that were available in CANTATAdb 2.0, such as expression levels and similarity search results against databases of lncRNAs and proteins, there are two major novelties in the current version. First, there are 112,980 lncRNAs that are expressed specifically in a particular plant part or embryo. Although we are not investigating their molecular functions, this information points to their possible organ- or developmental stage–specific roles in plants. The second novelty is data regarding evolutionarily conserved lncRNAs, which were identified using two approaches. Firstly, they were inferred from whole-genome alignments, based on the conservation of the genomic location (synteny), which does not require the two conserved counterparts to display detectable sequence similarity. In total, CANTATAdb 3.0 stores 74,886 pairs of these conserved lncRNAs found in 47 species; due to the resource-intensive nature of the associated computations, we were unable to provide the information for a wider range of species, as this would require the generation of over 10,000 genome alignments. Therefore, to complement this approach, we have also identified conserved lncRNAs using a reciprocal BLAST search, which was applied to all species pairs included in the database.

**Table 1:** A dissection of CANTATAdb 3.0 species into Magnoliopsida and seven other classes

Class	Families	Number of species	Number of lncRNAs
Magnoliopsida (flowering plants)	34* (see **Supplementary Table S1**)	100	580,557
Charophyceae (charophyte green algae; stoneworts and brittleworts)	Characeae	1	3,514
Chlorophyceae (unicellular green algae)	Chlamydomonadaceae	1	906
Florideophyceae (multicellular red algae)	Gigartinaceae	1	109
Bangiophyceae (red algae)	Galdieriaceae, Cyanidiaceae	2	2,655
Marchantiopsida (a class of liverworts)	Marchantiaceae	1	1,225
Bryopsida (the largest class of mosses)	Funariaceae	1	4,405
Lycopodiopsida (vascular plants: lycopods or lycophytes)	Selaginellaceae	1	2,317

### Database composition and usage

#### Search page

**Fig. 1:**
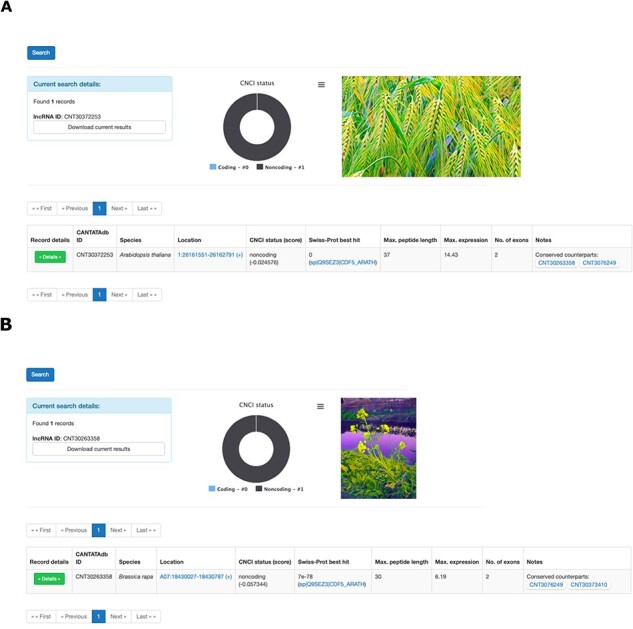
Screenshots of example CANTATA3.0 search results. (A) A snapshot of the entry of *A. thaliana* FLORE (CNT30372253) lncRNA, a NAT regulating expression of its sense CDF5 gene. According to Henriques et al., the CDF5/FLORE NAT pair constitutes a circadian regulatory module, able to adjust the onset of flowering to favorable environmental conditions (Henriques et al. 2017). Henriques et al. uncovered putative CDF/lncCDF NAT pairs in other species (e.g. *B. napus*) as well, suggesting evolutionary conservation of the regulatory mechanism. (B) A snapshot presenting FLORE’s ortholog in *B. rapa* (CNT30263358 entry).

To access the data stored in CANTATAdb 3.0, users must first select a species from the drop-down menu. Once selected, a search page is displayed, which includes the following components:

- Search Options: Users can specify search criteria and filters, including CANTATAdb lncRNA ID, Coding-Non-Coding Index (CNCI) status (coding or non-coding), length of the longest peptide within an lncRNA, organ-specific expression, conservation status, maximum expression value, number of exons and sorting preferences for the results.
- Search Summary: This section displays user-defined search parameters and the total number of matching records. Users can download the filtered data as a tab-delimited text file by clicking on the ‘Download Current Results’ button. In addition, a pie chart provides a visual summary of the distribution of CNCI status among the filtered lncRNAs.
- Search Results: Results are presented in a table format, with each row representing one lncRNA. Information displayed includes the lncRNA ID, species, genomic location, best match against Swiss-Prot proteins (if available), maximum peptide length (if applicable), maximum expression value across utilized RNA-Seq libraries used (in transcripts per million (TPM)), number of exons and notes, i.e. presence of conserved counterparts or plant part–specific expression (**Fig. 1**). In addition, there is a ‘Details’ button that, when selected, gives the user user access to further information about the selected lncRNA, including (a) the sequence of the lncRNA, (b) the results of BLAST searches against Swiss-Prot and lncRNAs for 21 plant species sourced from NONCODE v6, (c) a bar chart showing the expression profile of the lncRNA across the analyzed RNA-Seq libraries and (d) predicted peptide information associated with the lncRNA. From this detailed view, users can return to the search page, maintaining their search parameters, by clicking a button located at the top of the page.

#### BLAST page.

This section allows users to perform a sequence-based search within CANTATAdb 3.0 using BLAST version 2.2.26. A nucleotide sequence submitted by the user must comply with the FASTA format specifications. The user can choose between two tools, BLASTN and MEGABLAST. With the exception of the expectation value (*E*-value) and the maximum number of identified hits, the search parameters remain at their default settings, which are displayed on the same page for user convenience and reference.

#### Download page.

While the search results can be easily downloaded from the search page, a bulk data download function is also available. This allows users to obtain all lncRNAs separately for each species in both FASTA and general transfer format (GTF) formats. Additionally, the corresponding genome versions are presented for each species.

### Comparison with other databases

Considering the number of species analyzed, CANTATAdb 3.0 emerges as one of the most comprehensive repositories of plant lncRNAs currently available. For comparison, NONCODE v6 (Zhao et al. 2021) stores 94,697 lncRNAs from 23 plant species. The Plant Non-coding RNA Database 2.0 (Jin et al. 2021) contains lncRNAs from 80 plants, while PLNlncRbase (Xuan et al. 2015) contains manually collected data from nearly 200 publications, covering a total of 1,187 plant lncRNAs from 43 plant species. To the best of our knowledge, the most comprehensive databases of plant lncRNAs at the moment are currently GreeNC 2.0 (Di Marsico et al. 2022), which contains ∼500,000 putative lncRNAs from 94 plant and algal species, and JustRNA (Tseng et al. 2023), which contains 1,088,565 lncRNA annotations for 80 plant species. However, these platforms have their own drawbacks; for example, GreeNC has limited data download options and no lncRNA expression data and is not accessible at the time of writing (March 2024). There are also smaller, specialized or taxon-specific repositories. For example, AlnC (Singh et al. 2021) provides lncRNAs inferred from 1,000 plant (1KP) trancriptomes data. The current version of AlnC offers 10,855,598 annotated lncRNA transcripts from 682 species, but is limited to angiosperms, and lacks expression or lncRNA conservation data. In addition, EVLncRNAs 2.0 (Zhou et al. 2021) collects manually curated functional lncRNAs validated by low-throughput experiments; lncPheDB (Lou et al. 2022) sorted out a total of 203,391 lncRNA sequences, 2,000 phenotypes and 120,271 variants from nine plant species; RiceLncPedia (Zhang et al. 2021) collects rice lncRNAs. Finally, Ensembl Plants (Yates et al. 2022) catalogs lncRNAs in *Arabidopsis thaliana*, while the number of lncRNAs available for the other plant species is low (if any), e.g. one in *Arabis alpina*, 19 in *Populus trichocarpa* and none in *Hordeum vulgare*.

To better investigate how CANTATAdb 3.0 compares to similar databases, we performed one-to-one comparisons with selected repositories. By matching the coordinates of *A. thaliana* lncRNAs between Ensembl Plants 56 and CANTATAdb 3.0, we found 4,065 common lncRNAs, indicating that the majority of the 6,775 lncRNAs in CANTATAdb 3.0 overlap with this resource.

For GreenC 2.0, the lncRNA data are only available as FASTA sequences, so to compare them with CANTATAdb 3.0, we performed a similarity search using BLASTN. Only 11 out of 2,254 lncRNAs seemed to be the same in both resources, which is surprising because of the relatively large overlap between CANTATAdb 3.0 and Ensembl Plants. We cannot fully explain this discrepancy, but we have looked carefully at the data and identified a number of records with sense/antisense orientation, meaning that GreenC stores some lncRNAs in a potentially incorrect (reverse complement) orientation. We used a similar approach for the comparison with NONCODE v6, which stores 4,046 *A. thaliana* lncRNAs—1,018 of which were also found in CANTATAdb 3.0. However, the number doubles if the relative orientation between the query and the subject in the BLASTN search is disregarded, again suggesting problems in providing the correct lncRNA sequence; we confirmed this observation with BLASTN searches on the National Center for Biotechnology Information website using NONCODE lncRNAs as a query, which provided hits to known and annotated lncRNAs, yet again in a reverse complement orientation. This is alarming because many lncRNAs overlap with protein-coding genes on the other DNA strand, so database users may occasionally get a protein-coding sequence instead of lncRNAs.

In summary, CANTATAdb stands out from similar resources because of its comprehensive scope, including species whose lncRNAs cannot be found elsewhere. The quality of the data is ensured by a strict and previously published protocol for lncRNA identification, with the possibility of adding further filters via the database’s web interface. In addition, CANTATAdb provides valuable information such as conserved lncRNAs inferred from genome**–**genome alignments or reciprocal BLAST search results. Furthermore, the resource includes expression and tissue specificity data and has demonstrated stability through its third release, while some resources have never been updated or are currently unavailable. CANTATAdb provides easy access to the data in both FASTA and GTF formats, catering for different purposes, and also includes a BLAST search functionality.

### Tissue specificity as a hint toward possible functionality

One of the novelties introduced in CANTATAdb 3.0 is that lncRNAs are assigned to specific plant parts (roots, leaves, flowers, fruits, stems, anthers and seeds) as well as to an embryo. Data are presented for 80 species, depending on data availability. For example, for *Solanum lycopersicum*, there are 1,198 tissue-specific lncRNAs (fruit: 410, stem: 40, leaf: 359 and root: 389). Detailed statistics for each species can be found in **Supplementary Table S2**.

The fact that an lncRNA is shown as specific to a given plant part does not mean that it is not expressed in the other organs, but rather informs that its expression is elevated, with adjusted *P* values < 0.05, compared to the other plant parts. For example, CNT30509847 from *S. lycopersicum*, which is classified as fruit specific, is expressed at an average of 1,573 TPM in five fruit samples, while its average expression in the remaining 33 samples drops to 58.4 TPM. This clearly indicates that either the given lncRNA is actively regulated to play a role in the plant’s fruit or its expression is simply a by-product of the other regulatory processes, leading to organ-specific gene expression. Of note, multiple organ-specific lncRNAs as well as lncRNAs displaying organ-specific functions have already been described. For example, the MARS lncRNA, which shows a high tissue specificity in root tissues, plays a role in chromatin conformational changes in response to ABA (Roulé et al. 2022). Another lncRNA enriched in root tissue is At4, which has been shown to affect the phosphate distribution between the roots and the shoots during phosphate starvation (Shin et al. 2006).

### Conserved plant lncRNAs display elevated tissue specificity

**Fig. 2:**
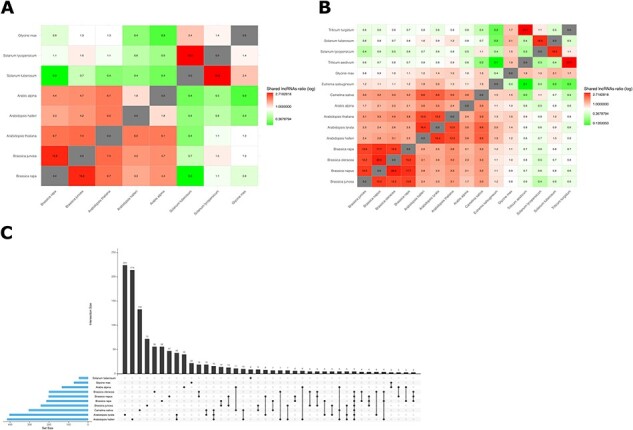
(A) A heatmap of syntenic lncRNAs shared between species pairs, as a fraction of all lncRNAs discovered in a given species (natural logarithm scale). (B) A heatmap for conserved lncRNAs obtained using reciprocal BLAST (natural logarithm scale); as conserved lncRNAs obtained using this approach span a larger set of species, the plot involves more representatives of Brassicaceae compared with the first heatmap. (C) An upset plot for conserved lncRNAs intersections, demonstrating the numbers of lncRNAs conserved between two or more species.

As lncRNAs tend to be poorly conserved in terms of their sequence, we generated whole-genome alignments that enabled search for conserved lncRNAs that occupy syntenic loci. Due to computational cost, the procedure was limited to a set of 1,286 genome pairs (**Supplementary Table S3**). In total, 24,270 lncRNAs from CANTATAdb have information about possible orthologs, corresponding to 74,886 orthologous pairs, as lncRNAs tend to have counterparts in more than one species. Typically, more than 5% of lncRNAs have orthologous lncRNAs with closely related species, e.g. *Brassica juncea* has 1,192 lncRNAs conserved in *B. rapa*, which corresponds to 13.46% of its lncRNA set. However, as expected, the numbers are drastically lower for distant species, e.g. only 0.3% of *S. tuberosum* lncRNAs are shared with *Oryza punctata* (**Fig. 2A**). It is important to note that our methodology captures novel as well as some known lncRNAs, such as COOLAIR, critical for controlling the expression of *FLOWERING LOCUS C* in plant development (Hawkes et al. 2016).

In order to provide the data across a broader spectrum of species pairs, we additionally employed a reciprocal BLAST search, yielding conserved lncRNAs across 5,479 species pairs (**Supplementary Table S4**). It is worth noting that, unlike the synteny-based approach, this procedure is not affected by errors in genome assembly and genome fragmentation that would yield suboptimal genome alignments. Moreover, it is not compute intensive, allowing us to apply it to the entire lncRNA spectrum in CANTATAdb 3.0. On the other hand, as mentioned earlier, the methodology is unable to identify conserved lncRNAs if sequence similarity has been lost, which might be a frequent phenomenon due to the generally poor conservation of lncRNAs at the sequence level (Deng et al. 2018, Szcześniak et al. 2021). Additionally, sequence similarity does not always reflect evolutionary relationships, for instance, if the aligned regions encompass repetitive elements. Last but not the least, it might be more prone to falsely classifying paralogous (not orthologous) genes as conserved counterparts. Nonetheless, a heatmap of species-to-species similarities demonstrates that the identified conserved lncRNAs generally reflect the taxonomic relationships (**Fig. 2B**), with more conserved lncRNAs identified between evolutionarily close species, just like with syntenic lncRNAs. To better understand the pattern of lncRNA conservation, we have prepared an upset plot for Brassicaceae (**Fig. 2C**), which displays the size of intersections (i.e. common lncRNAs) between *A. thaliana* and other species. As seen in the plot, the vast majority of lncRNAs are only shared between two species, pointing to their poor conservation, with the largest number of lncRNAs uniquely shared with *A. lyrata* (227), followed by *A. halleri* (217). At the same time, *A. halleri* and *A. lyrata* together have the highest number of lncRNAs conserved in *A. thaliana* (46), reflecting their close evolutionary relationship.

With lists of tissue-specific lncRNAs, we wanted to check whether conserved lncRNAs tend to have elevated expression levels in the same plant parts; for example, are orthologs of *A. thaliana* root-specific lncRNAs also predominantly root specific in *A. halleri*? We therefore calculated the percentage of conserved lncRNAs that are specific to the same plant part (e.g. if 12 root-specific lncRNAs in species A are also root specific in species B, out of 50 orthologs, the percentage is 24%). This was compared with the percentage of all lncRNAs assigned to the given plant part (e.g. if 500 lncRNAs out of 5,000 were predicted to be root specific, this is 10%). A chi-square test showed that conserved lncRNAs do indeed tend to be assigned to the same body part: in 53 out of 59 species pairs (89.8%) with syntenic and organ-specific lncRNAs, the *P* value was below 0.05; in the case of reciprocal BLAST–derived conserved lncRNAs, the *P* value was below 0.05 in 488 species pairs (94.0%). To get a deeper insight into the issue, we calculated tissue specificity Tau scores. As expected, lncRNAs have a significantly higher tissue specificity than protein-coding genes (Mann–Whitney, *P* value < 1e-5; **Supplementary Fig. S1**). Further analysis showed that all lncRNAs with their conserved counterparts generally have higher Tau scores, compared to the remaining CANTATAdb lncRNAs, (Mann−Whitney, *P* value < 1e-5, syntenic lncRNAs). Both pieces of evidence associate tissue-specific or organ-specific expression with increased evolutionary conservation of plant lncRNAs, which is somewhat surprising, as higher tissue specificity of lncRNAs has been associated with lower evolutionary conservation (Trizzino et al. 2018, Szcześniak et al. 2019); in particular, there are many human-specific lncRNAs with high tissue specificity, in contrast to broadly expressed protein-coding genes (Szcześniak et al. 2019). Plants, however, have a significantly lower number of lncRNAs, which could be the result of a stronger purifying selection to maintain functional lncRNAs; in this light, the tissue-specific or organ-specific lncRNAs could be those with the established function, explaining their increased evolutionary conservation.

### Final conclusions

The updated CANTATAdb 3.0 database is a comprehensive resource for plant lncRNAs, covering a relatively wide range of species and providing valuable insights into their biology, including expression patterns and conservation. The presented organ-specific and conserved lncRNAs help to highlight lncRNAs with potential roles in plant biology. Interestingly, our analysis revealed that conserved lncRNAs tend to show expression restricted to the same plant organs, suggesting conserved functions across species.

CANTATAdb 3.0 contributes to a better understanding of the diversity and potential roles of plant lncRNAs, facilitates further research in this field with its user-friendly interface and search tools and promises to be a valuable resource for exploring the regulatory functions, evolutionary dynamics and potential applications of plant lncRNAs in biology and biotechnology.

## Materials and Methods

### Ab initio transcriptome assembly

The RNA-Seq reads in FASTQ format were subjected to quality filtering and adapter trimming using BBDuk v37.02 (https://jgi.doe.gov) with the following settings: qtrim = w, trimq = 20, maq = 10, rref = bbmap/resources/adapters.fa, k = 23, mink = 11, hdist = 1, tbo, tpe, minlength = 50 and removeifeitherbad = t. Reads from a given species were then mapped against the corresponding plant genome using STAR v2.5.3a (Dobin et al. 2013). STAR settings are as follows: --outSAMattributes All, --outSAMattrIHstart 0, --outSAMtype BAM Unsorted, --outSAMunmapped Within, --outSAMstrandField intronMotif, --outFilterIntronMotifs RemoveNoncanonical, --outFilterType BySJout, --outFilterMultimapNmax 20, --alignSJoverhangMin 8, --alignSJDBoverhangMin 1, --outFilterMismatchNmax 999, --outFilterMismatchNoverLmax 0.04, --alignIntronMin 20, --alignIntronMax 100000, --alignMatesGapMax 100000, --twopassMode Basic, --chimSegmentMin 12, --chimJunctionOverhangMin 12 and --chimSegmentReadGapMax 3. The resulting BAM file was then sorted using samtools sort and used for ab initio transcriptome assembly with StringTie (Pertea et al. 2015), where downloaded annotations in GTF format served as a reference. This generated a new GTF file with a custom transcriptome, one per sample. To get a single, consensus transcriptome per species, the transcriptomes were merged with StringTie, using ‘--merge’ parameter. This resulted in a single GTF file that was further subjected to filtering. First, transcripts with no strand assigned were discarded. Then, the transcriptome was compared against the reference transcriptome from Ensembl Plants 56 and transcripts with selected class codes (‘c’, ‘e’, ‘p’ and ‘s’) were discarded, as they represent potential artifacts in the transcriptome assembly. Transcript sequences in FASTA format were extracted from the plant and algae genomes, taking advantage of GTF files from ab initio assembly and using the gffread utility from Cufflinks package v2.2.1 (Trapnell et al. 2012).

### Identification of lncRNAs

The annotations from the GTF file were compared with the reference annotations using Cuffcompare from the Cufflinks package, with -R (‘considers only the reference transcripts that overlap any of the input transfrags’) and -C (‘includes the “contained” transcripts in the combined gtf file’) options. The identification of lncRNAs was then performed in the following steps, as implemented in in-house Python scripts:

- Discarding transcripts with Cuffcompare class codes *=, j, o* if reference transcripts were not classified as lncRNAs at Ensembl (‘rRNA’, ‘IG_C_pseudogene’, ‘protein_coding’, ‘IG_V_gene’, ‘polymorphic_pseudogene’, ‘misc_RNA’, ‘scaRNA’, ‘IG_J_gene’, ‘TR_J_pseudogene’, ‘IG_J_pseudogene’, ‘TEC’, ‘Mt_tRNA’, ‘Mt_rRNA’, ‘TR_V_pseudogene’, ‘TR_J_gene’, ‘TR_D_gene’, ‘IG_V_pseudogene’, ‘nonsense_mediated_decay’, ‘snRNA’, ‘sRNA’, ‘TR_V_gene’, ‘miRNA’, ‘IG_C_gene’, ‘ribozyme’, ‘IG_D_gene’, ‘TR_C_gene’, ‘snoRNA’, ‘vaultRNA’ and ‘non_stop_decay’).
- Discarding transcripts shorter than 200 bases.
- Open reading frames (ORFs) were identified using TransDecoder 5.0.2 (https://github.com/TransDecoder/TransDecoder) with the -S (‘strand-specific’) option. If the predicted protein was longer than 100 amino acids, the candidate was discarded, regardless of whether it contained start and stop codons. Any ORFs that produced proteins longer than 50 AA, containing both start and stop codons and displaying similarity to Viridiplantae proteins from Swiss-Prot (BLASTP, *E*-value < 1e-5), were also discarded.
- Discarding transcripts classified as ‘coding’ by Coding Potential Calculator (CPC) v0.9-r2 (Kong et al. 2007) with default settings.
- A BLASTN v2.2.26 search was performed against sequences from the Rfam database (Kalvari et al. 2018), and all sequences with hits of *E*-value < 1e-5 against subjects described as ‘miRNA’, ‘microRNA’, ‘snoRNA’, ‘snRNA’, ‘tRNA’, ‘small nucleolar RNA’, ‘small nuclear RNA’, ‘rRNA’ or ‘ribosomal RNA’ were removed.

Regardless of the TransDecoder and CPC results, all RNAs classified into the following lncRNA-related Ensembl transcript types were retained: ‘retained_intron’, ‘sense_intronic’, ‘sense_overlapping’, ‘antisense_RNA’, ‘antisense’, ‘lincRNA’, ‘macro_lncRNA’, ‘bidirectional_promoter_lncRNA’, ‘lncRNA’ and ‘lncRNAs’.

### Annotation of the identified lncRNAs

To expand our understanding of the identified lncRNAs, we performed additional analyses. First, we used CNCI (Sun et al. 2013) in a plant-specific mode (with the ‘model’ parameter set to ‘pl’) to assess the coding potential of all lncRNAs, independent of CPC. Subsequently, using TransDecoder, we detected potential peptides and proteins originating from the sense strand of lncRNAs. For this purpose, we used a parameter (-m 30) that allows the identification of ORFs that are at least 30 amino acids long, whereas the default threshold is 100 amino acids. In this analysis, the presence of start or stop codons within ORFs was not mandatory. Once the ORFs were identified, we conducted a BLASTP v2.2.26 search against a non-redundant and manually curated dataset of Viridiplantae proteins sourced from the Swiss-Prot database (The UniProt Consortium 2023). In addition, we performed a BLASTX search against Swiss-Prot proteins (confined to Viridiplantae), using an *E*-value threshold of 1e-5, to ascertain any sequence similarities between lncRNAs and known proteins, including regions beyond the identified ORFs. Subsequently, a BLASTN search was performed against NONCODE v6 lncRNAs, spanning 21 species, with parameters set to -e 1e-5 and -F F (for filtering out low-complexity regions in queries). Expression estimation was carried out using SALMON (Patro et al. 2017) in a quasi-mapping mode, with ‘seqBias’ and ‘gcBias’ parameters to correct for biases due to nucleotide composition within the transcript sequences. For tissue specificity calculations, we used Tau metrics (Yanai et al. 2005), which have previously been shown to perform well compared with other tissue specificity measures (Kryuchkova-Mostacci and Robinson-Rechavi 2017). In the calculations, gene expression estimations in TPM units were used.

### Search for evolutionarily conserved lncRNAs

The evolutionarily conserved lncRNAs were searched in two modes, with synteny and using the similarity search approach. For the former, plant genome sequences were downloaded from Ensembl Plants 56 and their FASTA index was obtained using samtools faidx (Li et al. 2009). A given pair of plant genomes was aligned with *nucmer* utility of MUMmer4 (Marçais et al. 2018), using the following non-default parameters for increased sensitivity: ‘mincluster 50’ (the minimum length of a cluster of matches), ‘minmatch 15’ (the minimum length of a single exact match) and ‘breaklen 300’ (the distance an alignment extension will attempt to extend poorly scored regions before giving up). The resulting alignments in a delta format were then converted to a chain format using the *crossmap_delta_to_chain.pl* script from crossmap workflow (https://github.com/soybase/crossmap-workflow). The chain files were then subjected to sorting, netting and cleaning, with *chainSort, chainNet* and *chainCleaner* utilities, respectively; all of them come from Genome Alignment Tools (https://github.com/hillerlab/GenomeAlignmentTools; Kent et al. 2003) and were applied with default settings. Finally, the genome alignments were used as input to slncky (Chen et al. 2016) to identify lncRNAs that are conserved between the two species of interest. The following parameters were used: ‘--no_filter’ (the lncRNAs are not subject to filtering, i.e. the input dataset remains complete) and ‘--no_orf’ (switching off the search for ORFs). In addition, the analysis requires lncRNAs and protein-coding gene annotations in a BED format, which are obtained by processing GTF files with a custom Python script. In the output of slncky, only the so-called ‘top’ results were considered, i.e. a single, best-scoring hit per lncRNA.

In the similarity search approach, a reciprocal BLAST search was employed. Here, a BLASTN search was performed, with a full list of CANTATAdb 3.0 lncRNAs serving both as a query and a target database and an *E*-value threshold of 1e-5. Using a Python script, the BLASTN results were filtered to keep only cases with at least 20% coverage (i.e. alignment length constituted at least 20% of target or query length). We have applied a reciprocal BLAST search here. Briefly, this involved running a BLAST search of a query lncRNA against a target database (lncRNAs from another species) to find the best match (with lowest *E*-value) and then using that best match as a new query to search against the original database. If the top hit in this reverse search matched the original query sequence, the sequences were considered conserved lncRNAs.

### Organ-specific lncRNAs

For each species, plant body parts were identified with at least three replicates. This resulted in sequencing libraries in 78 plants representing the following plant parts: root, flower, fruit, stem, anther, leaf and seed. The list was complemented with a plant embryo. The selection was made manually based on sample and run descriptions at the Sequence Read Archive. For example, samples described as being from ‘leaf’, ‘leafs’ and ‘leaves’ were included in the ‘leaf’ category. At the same time, any changes, such as ‘leaf bud’, mixed samples, e.g. ‘leaf and stem’, as well as any erroneous or dubious cases (e.g. a sample described as ‘root’, but the corresponding sequencing runs are assigned to a ‘leaf’) were avoided. The selected replicates, called ‘target samples’, were then subjected to differential gene expression analysis of genes, by comparing them with all other samples (‘background samples’) in a given plant species. Here, dubious samples or those with no source assigned (‘NA’) were discarded to avoid a possible scenario of comparing samples originating from the same source. In this task, DESeq2 was used; an lncRNA was considered to be expressed specifically in a given plant part or embryo if the adjusted *P* value was below 0.05 and its expression was elevated in the ‘target samples’. Here, lncRNA genes were subject to differential expression analysis, not individual transcripts. Importantly, we generated diagnostic plots, in particular, sample clustering using principal component analysis, which occasionally indicated that selected ‘background samples’ were close to ‘target samples’. For example, samples representing ‘shoots’ (plant stem with leaves) typically clustered with ‘leaves’ samples; such cases were discarded.

### Database implementation and testing

The database was built using Hypertext Markup Language, Cascading Style Sheets, PHP 8.1.27 (http://www.php.net/), MySQL 8.0.36 (http://www.mysql.com/) and a Bootstrap 3.3.4 (http://getbootstrap.com/) framework. The database layout has also been enhanced with the JavaScript Highcharts library (http://www.highcharts.com/) for interactive data plotting.

The web interface and database functionality have been tested on Windows and Mac OS X operating systems using Edge (versions 110–118), Mozilla Firefox (versions 110–119), Chrome (versions 110–119), Opera (versions 100–104) and Safari (versions 5.0–5.1.5) web browsers.

## Supplementary Material

pcae081_Supp

## Data Availability

The data underlying this article are available within the article and in the online database: http://cantata.amu.edu.pl/ and http://yeti.amu.edu.pl/CANTATA/.
