# Supplementary figures and images for: CANTATAdb 3.0: An Updated Repository of Plant Long Non-Coding RNAs
Source: Plant Cell Physiol. 2024 Jul 17;65(9):1486–93. doi: 10.1093/pcp/pcae081 (PMC11447640; doi:10.1093/pcp/pcae081)

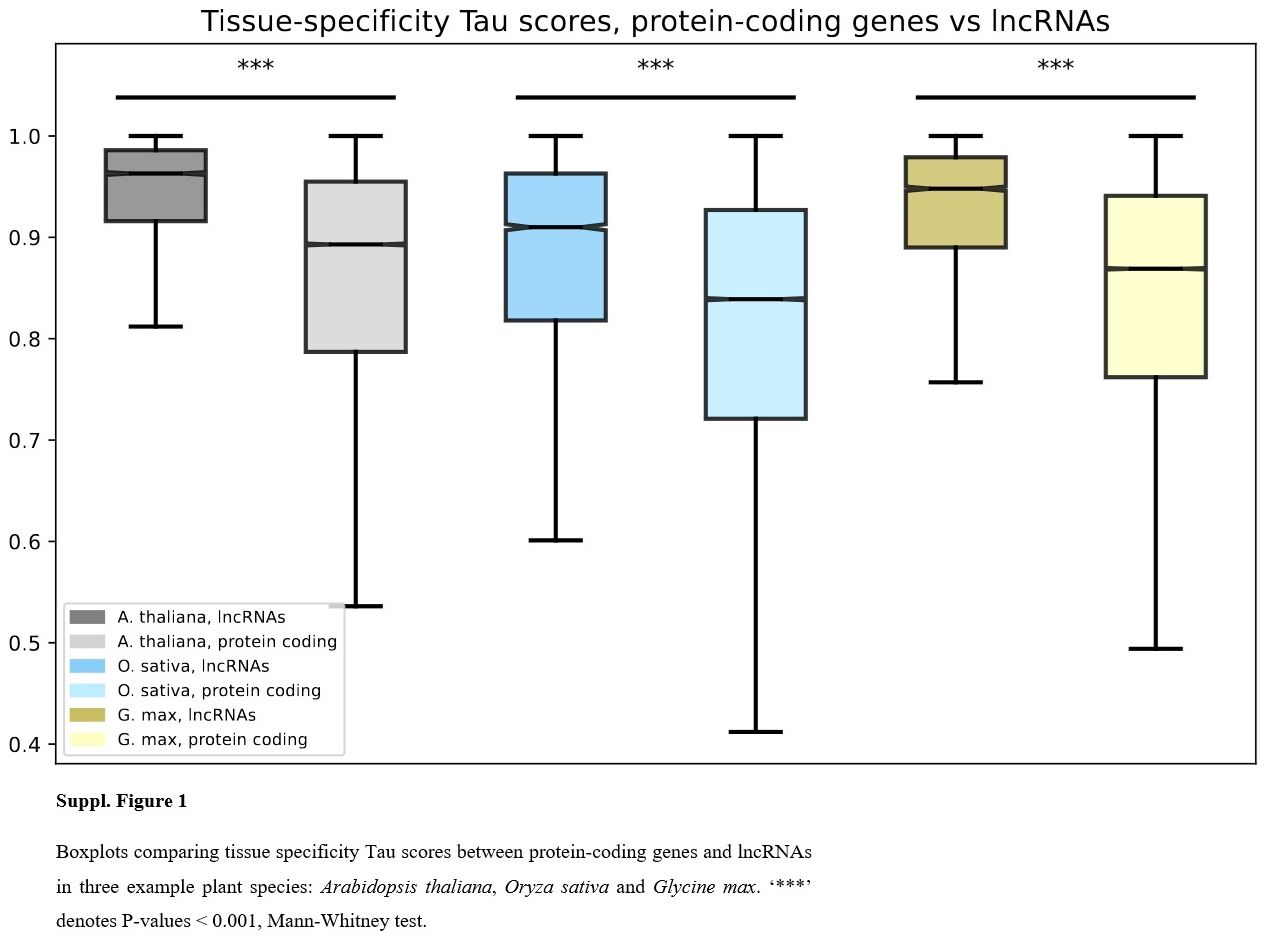

Supplement: pcae081_Supp [file pcae081_supp.zip › suppl_data/pcp-2024-e-00081-File009.jpg]
